# Telepharmacy for Chronic Disease Management in Saudi Arabia Compared With the United States and the United Kingdom: A Narrative Review

**DOI:** 10.7759/cureus.99836

**Published:** 2025-12-22

**Authors:** Abdullah Alali, Faisal Nasser, Mohammed Bin Rakan, Marwan A Alrasheed

**Affiliations:** 1 Department of Clinical Pharmacy, College of Pharmacy, King Saud University, Riyadh, SAU

**Keywords:** chronic disease, electronic prescribing and medicines administration (epma), kingdom of saudi arabia (ksa), pharmacy-led telehealth, remote medication management, telepharmacy, united kingdom, united states of america, virtual pharmaceutical care

## Abstract

Telepharmacy is increasingly recognized as a transformative model for improving access to pharmaceutical care, enhancing and supporting chronic disease management through remotely delivered medication review, therapy optimization, and adherence support. By enabling structured pharmacist-led interventions, telepharmacy has the potential to enhance medication safety by reducing prescribing errors, improving monitoring of high-risk therapies, and strengthening continuity of care across healthcare settings. Saudi Arabia has rapidly expanded digital health initiatives under Vision 2030, yet its telepharmacy landscape remains in an evolving developmental stage compared with mature systems in the United States of America (USA) and the United Kingdom (UK). This narrative review examines the enabling and limiting factors influencing telepharmacy implementation for chronic disease management across these three regions, with a focus on digital infrastructure, clinical outcomes, regulatory preparedness, pharmacist readiness, and patient experience. Saudi Arabia demonstrates early clinical success and features advanced institutional models. However, persistent challenges include regulatory ambiguity, inconsistent enforcement, limited pharmacist training, digital literacy gaps, and a shortage of rigorous outcome-based research. Insights from the USA and UK emphasize the importance of national governance, sustainable reimbursement models, and standardized workflows. Strengthening regulation, enhancing workforce training, and establishing financial sustainability are critical for advancing telepharmacy within Saudi Arabia's chronic disease management pathways.

## Introduction and background

Telepharmacy, defined as the remote provision of pharmaceutical care using telecommunication technologies, has emerged as a critical tool for supporting medication counseling, therapeutic monitoring, medication therapy management (MTM), and chronic disease follow-up [1]. Its adoption continues to grow globally as healthcare systems expand digital services to manage the rising burden of chronic diseases. In advanced healthcare systems such as those of the United States of America (USA) and the United Kingdom (UK), telepharmacy is widely deployed across outpatient, community, hospital, and specialty settings, where it has demonstrated consistent improvements in medication adherence, clinical outcomes, and patient satisfaction [1, 2].

In the United States, pharmacist-led telehealth is supported by a strong digital infrastructure and established reimbursement pathways, allowing for widespread integration into hospital and community settings [2]. Similarly, in the United Kingdom, telepharmacy is deeply embedded within the centralized National Health Service (NHS) digital infrastructure, supported by national e-prescribing and virtual medication optimization services [3]. These mature models offer valuable case studies in governance, workflow standardization, and interdisciplinary coordination.

Saudi Arabia has paralleled this global trend, rapidly expanding its digital health ecosystem under the framework of Vision 2030 and the Health Sector Transformation Program [4, 5]. This digital transformation is supported by national platforms such as Sehhaty and the Wasfaty e-prescribing system, which have modernized access to care [6, 7]. Evidence from the Kingdom, primarily derived from observational and quasi-experimental studies, suggests the potential clinical benefits of these initiatives. For example, telemedicine-supported diabetes programs involving adult patients with uncontrolled type 2 diabetes have reported improvements in glycemic control over short- to medium-term follow-up periods [8]. Similarly, pharmacist-led remote anticoagulation monitoring clinics, evaluated using retrospective cohort designs, have demonstrated safer anticoagulation management and comparable therapeutic outcomes to in-person services [9]. Furthermore, advanced institutional models, such as the ambulatory telepharmacy services at Johns Hopkins Aramco Healthcare (JHAH), highlight the potential for high-maturity implementation within the region [10].

Despite the rapid expansion of digital health initiatives, a critical gap remains in the current literature. The majority of existing studies on telepharmacy in Saudi Arabia have predominantly focused on the knowledge, perception, and readiness of healthcare professionals [11, 12]. There is a notable scarcity of research that rigorously evaluates clinical outcomes, regulatory frameworks, or comparative system performance against established international standards. Furthermore, few studies have synthesized the lessons learned from mature telepharmacy markets to offer actionable policy recommendations for the Kingdom's evolving healthcare landscape.

To address this gap, this narrative review benchmarks Saudi Arabia’s telepharmacy progress against the USA and the UK. These two nations were selected as comparators because they represent the dual nature of Saudi Arabia's healthcare transformation. The UK’s NHS, which predates the establishment of Saudi Arabia’s modern Ministry of Health infrastructure, provides a model of a centralized, publicly funded healthcare system. This model offers valuable insights into national governance and standardized digital workflows. Conversely, the USA offers a mature model of a privatized, insurance-based system, which aligns closely with Saudi Arabia’s current strategic shift toward privatization and corporatization [5]. By analyzing these distinct models, this review aims to provide a comprehensive roadmap for navigating the transition from a state-run to a value-based, digitally integrated pharmaceutical care system.

## Review

Methods

A structured narrative review was conducted to examine telepharmacy implementation, remote pharmaceutical services, and chronic disease management across Saudi Arabia, the USA, and the UK. Searches were performed in PubMed (including Medical Literature Analysis and Retrieval System Online (MEDLINE)) and Scopus for studies published between January 2015 and October 2025. This timeframe was selected to capture contemporary evidence generated during key digital health reforms, including Saudi Arabia’s Vision 2030 and the expansion of national e-health platforms such as Wasfaty and Sehhaty. The search strategy used combinations of keywords and Boolean operators, incorporating terms such as “telepharmacy,” “telehealth,” “telemedicine,” “pharmaceutical care,” “medication,” “Saudi Arabia,” “United States,” “United Kingdom,” and “NHS.” Relevant governmental and organizational reports were also reviewed to supplement the regulatory and policy context.

After duplicate records were removed, titles and abstracts were screened to identify potentially relevant publications. A total of 73 reports were sought for full-text retrieval, of which 17 could not be obtained due to access limitations or unavailable PDFs. The remaining 56 full-text articles were assessed for eligibility. Studies were included if they described pharmacist-led remote care, telepharmacy services, or digital medication management; addressed chronic disease management; were conducted in Saudi Arabia, the USA, or the UK; and were published in English. Full-text reports were excluded if they did not involve telepharmacy or remote pharmaceutical services, did not focus on chronic disease management, were conducted outside the defined countries, or did not address medication-related outcomes or aspects of medication optimization. Following application of these criteria, 24 studies were included in the final synthesis (Saudi Arabia: 11; USA: 8; UK: 5).

Data from the included studies were extracted narratively and synthesized across four comparative domains: digital infrastructure, regulatory frameworks, workforce readiness, and clinical outcomes. As this review did not follow a systematic or meta-analytic methodology, no formal risk of bias assessment was performed; however, key methodological characteristics of the included literature were considered to contextualize the strength of the evidence.

**Figure 1:**
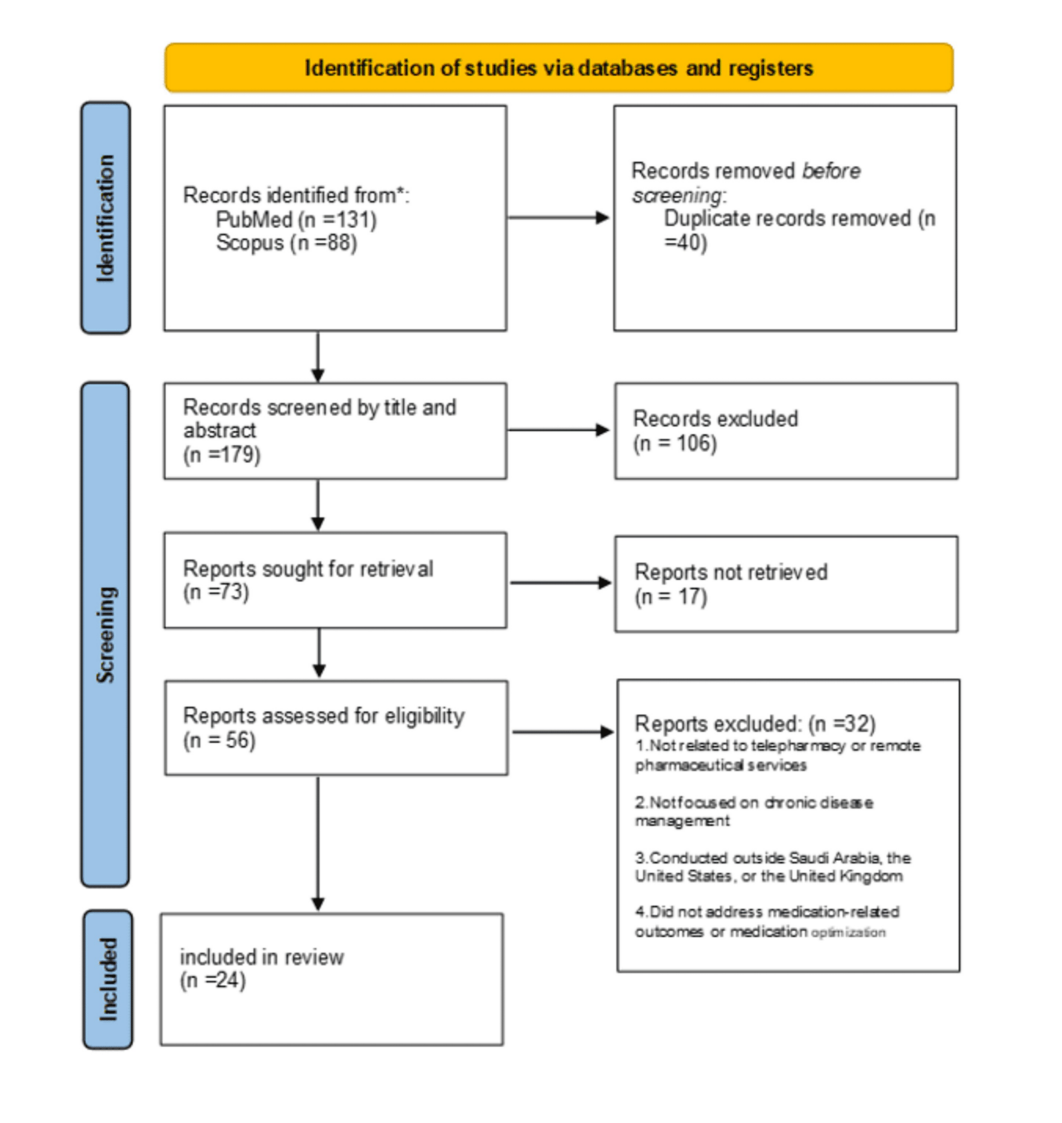
A PRISMA flow diagram illustrating the identification, retrieval, screening, and inclusion of studies for this narrative review. The diagram is adapted from PRISMA 2020 guidelines to enhance transparency in the study selection process. PRISMA: Preferred Reporting Items for Systematic Reviews and Meta-Analyses

The overall process of study identification, retrieval, screening, and inclusion is summarized in Figure 1, which presents a Preferred Reporting Items for Systematic Reviews and Meta-Analyses (PRISMA) 2020 flow diagram adapted for this narrative review.

Results

Telepharmacy Landscape in Saudi Arabia

Saudi Arabia has achieved a rapid digital transformation in healthcare, driven by high adoption rates of e-health tools among professionals. The Kingdom’s model is centralized, relying on government-led platforms such as Sehhaty for patient engagement and Wasfaty for electronic prescribing [6, 7]. Quantitative clinical evidence from the Kingdom demonstrates the effectiveness of medication-focused remote care models in selected chronic conditions. In a prospective pre-post telemedicine intervention study involving 130 adults with uncontrolled type 2 diabetes (baseline HbA1c >9%), virtual integrated care was associated with a statistically significant reduction in HbA1c from 9.98 ± 1.33 to 8.32 ± 1.31 over a four-month follow-up period (mean reduction 1.66%, p < 0.001) [8]. More recent data from Seha Virtual Hospital indicate that centralized virtual interventions are feasible and effective for managing complex cases remotely [13]. Similarly, pharmacist-led telepharmacy anticoagulation services have been evaluated in a single-center retrospective cohort study including 191 warfarin-naïve patients, of whom 77 were followed via telepharmacy and 114 through conventional on-site clinics. The primary outcome, time in therapeutic range (TTR), was comparable between the telepharmacy and on-site groups (50% vs. 53.8%, p = 0.455), with no statistically significant differences in thromboembolic events, bleeding complications, or extreme international normalized ratio (INR) values. These findings support the non-inferiority of remote pharmacist-led anticoagulation management compared with standard in-person care within the Saudi healthcare context [9].

Despite these clinical successes, the operational landscape faces challenges. While the Wasfaty system has improved medication access, recent evaluations highlight critical safety gaps, including deficiencies in key functional features necessary for e-prescribing best practices, such as integrated clinical decision support [14]. Additionally, technical limitations such as a lack of real-time stock visibility disrupt the continuity of virtual care [7]. Furthermore, although community pharmacists express a willingness to adopt telepharmacy, they report significant gaps in digital competency and a lack of standardized remote counseling workflows [11]. Institutional exemplars, such as the ambulatory telepharmacy model at JHAH, demonstrate high maturity through integrated call centers and automated refilling systems [10], yet these advanced models remain the exception rather than the norm. Regulatory enforcement also remains inconsistent, with studies highlighting ambiguity regarding online pharmacy standards (Table 1) [12].

**Table 1:** Current landscape of telepharmacy in Saudi Arabia: enablers and barriers MOH: Ministry of Health; JHAH: Johns Hopkins Aramco Healthcare; TTR: time in therapeutic range; HbA1c: hemoglobin A1c

Domain	Current Infrastructure & Models	Key Challenges & Gaps
Digital platforms	National scale: Sehhaty (Patient interface) and Wasfaty (e-Prescribing) [6, 7]. Institutional: High-maturity systems at JHAH (e.g., MyChart, remote counseling) [10].	Operational: Lack of clinical decision support in Wasfaty [15]; stock visibility issues disrupting workflow [7].
Clinical outcomes	Success cases: Tele-diabetes programs showing reduced HbA1c [8] and virtual anticoagulation clinics maintaining TTR [9].	Evidence gap: Lack of data on other chronic conditions (e.g., hypertension, asthma); need for more rigorous outcome-based research [12].
Workforce	Attitude: Pharmacists report high willingness to engage in telepharmacy [11].	Competency: Significant gaps in digital health training and competency; lack of standardized workflows for remote counseling [11].
Patient experience	Access: High satisfaction with primary care telemedicine and positive user ratings [15].	Barriers: Digital literacy issues, particularly among older adults; concerns regarding privacy and usability [15].

Telepharmacy in the USA: A Decentralized, Reimbursed Model

The USA features a mature, decentralized telepharmacy ecosystem driven by private-sector innovation and established reimbursement pathways [2]. Unlike the centralized Saudi model, U.S. telepharmacy is heavily influenced by state-level regulations. A prime example is the North Dakota Model, which utilizes remote pharmacist verification to sustain pharmacy services in rural areas and has been replicated across multiple states [16]. Pharmacist-led telehealth is also integrated into hospital settings, with 28.4% of U.S. hospitals reporting the provision of telepharmacy services, reflecting institutional adoption of remote pharmaceutical care [2]. Crucially, the U.S. system supports financial sustainability through MTM billing codes (e.g., Current Procedural Terminology (CPT) codes 99605-99607), allowing pharmacists to be reimbursed for remote cognitive pharmaceutical services [17]. Clinical evidence from U.S.-based interventional studies demonstrates meaningful improvements in chronic disease outcomes associated with pharmacist-led telepharmacy. A rural pilot feasibility study reported reductions in HbA1c of 0.57% among patients with baseline HbA1c between 7% and 10% and 2.55% among patients with HbA1c ≥10%, while at least one drug therapy problem was identified in 44% of patients [18].

Additional interventional evidence supports telepharmacy for hypertension management. A pharmacist-led telehealth quality improvement initiative demonstrated a reduction in mean systolic blood pressure from 155.2 mmHg to 132.1 mmHg and diastolic blood pressure from 89.7 mmHg to 77.6 mmHg, with 31% of patients achieving guideline-recommended blood pressure targets (<130/80 mmHg) without compromising safety [19].

Beyond common chronic conditions, telepharmacy has been applied to complex care populations. A pragmatic randomized pilot study involving people living with dementia and polypharmacy demonstrated high interdisciplinary integration, with 98% of pharmacists’ medication recommendations accepted by primary care providers and 81.5% of patients experiencing deprescribing of at least one medication, alongside modest reductions in medication regimen complexity [20]. At a systems level, large-scale pharmacist-led telehealth initiatives, such as the STIC2IT (Study of a Tele-pharmacy Intervention for Chronic diseases to Improve Treatment adherence) trial, illustrate advanced operational maturity and scalability of telepharmacy within U.S. primary care, although outcome data were not reported in the design publication [21]. Systematic reviews confirm that these interventions improve adherence and chronic disease metrics such as blood pressure and glycemic control [22]. Specialty programs, such as those for kidney transplant recipients, have further demonstrated that telepharmacy can bridge gaps in complex postoperative care (Table 2) [23].

**Table 2:** Current landscape of telepharmacy in the United States: enablers and barriers EHR: electronic health record; CDSS: clinical decision support system; MTM: medication therapy management

Domain	Current Infrastructure & Models	Key Challenges & Gaps
Digital platforms	Decentralized systems with interoperable EHRs and CDSS across major health systems; rural telepharmacy supported through models such as the North Dakota Model [2, 16].	Significant variability in digital maturity and telepharmacy authorization across states [16].
Clinical outcomes	Demonstrated improvements in HbA1c, blood pressure, adherence, and deprescribing; widely integrated into hospital and primary care telehealth programs [18-21].	Outcomes differ by region; limited long-term data for some chronic conditions [22].
Workforce	Strong telehealth experience; telepharmacy training integrated into PharmD curricula and simulations; technicians support remote verification workflows [24].	Training inconsistent across settings; independent pharmacies may lack digital readiness [24].
Patient experience	High satisfaction and expanded access, especially in rural and underserved populations [18, 20].	Digital divide persists in low-income and rural areas; concerns about privacy and connectivity [18]

Telepharmacy in the UK: Integrated NHS Governance

In the UK, telepharmacy-related services are embedded within the centralized infrastructure of the NHS, offering a model of high governance and national standardization. Although the term “telepharmacy” is not routinely used across the UK, medication-focused remote pharmaceutical activities are delivered within broader NHS telehealth and medicines-optimization frameworks. The Electronic Prescription Service (EPS) serves as the national digital backbone, enabling standardized electronic prescribing across care settings [3].

A key distinction of the UK model is the formal integration of remotely deliverable pharmaceutical support into nationally commissioned care pathways. Initiatives such as the New Medicine Service (NMS) and the Discharge Medicines Service (DMS) enable community pharmacists to provide structured consultations, frequently conducted by telephone, to support patients initiating new therapies or transitioning from hospital to primary care [25,26]. Although these services are not labeled as “telepharmacy,” the remote consultation elements align with telepharmacy principles. The clinical effectiveness of NMS is supported by robust evidence; a pragmatic randomized controlled trial by Elliott et al. (2016) demonstrated that NMS improved medication adherence compared with usual care [27]. However, the longer-term follow-up study found that this improvement was not sustained at 26 weeks, with adherence differences no longer reaching statistical significance. This suggests that while early pharmacist-led follow-up delivered remotely can enhance short-term adherence, additional or ongoing support may be required to maintain benefits over time [28].

Direct evidence of telepharmacy implementation in the UK has also been demonstrated through a proof-of-concept study conducted in rural Scotland. This model, based on remote pharmacist supervision and clinical support, showed that telepharmacy can be a safe and acceptable method for maintaining access to pharmaceutical services in geographically isolated communities [29].

In addition, broader remote-care models have been evaluated within chronic respiratory disease management. Telehealth interventions for patients with chronic obstructive pulmonary disease (COPD), delivered through remote monitoring or virtual consultations, have demonstrated mixed clinical effects, with little to no consistent improvement in symptoms or quality of life. However, the Cochrane systematic review reported that such interventions may reduce COPD-related hospital readmissions and support the feasibility of remote care delivery within multidisciplinary NHS frameworks [30]. Additionally, international assessments of medication-review practices show that although the UK consistently delivers the essential elements of high-quality reviews, variations in follow-up and documentation indicate that further standardization could enhance the consistency and long-term effectiveness of remotely delivered pharmaceutical care across the NHS [31].

Overall, the centralized NHS infrastructure supports equitable access and standardized delivery of pharmaceutical services, though the level of digital engagement varies across regions and populations (Table 3).

**Table 3:** Current landscape of telepharmacy in the United Kingdom: enablers and barriers NHS: National Health Service; EPS: Electronic Prescription Service; NMS: New Medicine Service; DMS: Discharge Medicines Service; COPD: chronic obstructive pulmonary disease

Domain	Current Infrastructure & Models	Key Challenges & Gaps
Digital platforms	Centralized NHS digital infrastructure with nationwide EPS; remote support embedded in NMS and DMS pathways; rural telepharmacy proven feasible [3, 25, 26, 29].	Regional differences in digital capability; reliance on telephone consultations in some locations [25, 26].
Clinical outcomes	NMS improves short-term adherence; remote counseling supports therapy initiation and transitions; COPD telehealth reduces readmissions in some settings [27–30].	Long-term adherence benefits not sustained; mixed results in chronic respiratory diseases [27, 30].
Workforce	Structured pharmacist roles within NHS-commissioned services; strong technician integration; effective multidisciplinary collaboration [25, 26].	Workforce strain in community settings; variable training in digital consultation skills [26].
Patient experience	High accessibility through NHS-funded services; strong acceptance among older adults; reduced travel burden [27].	Digital literacy gaps and preference for in-person care among some groups [28].

Comparative Analysis

Governance and regulatory frameworks: A comparative assessment reveals fundamental structural differences in how telepharmacy is governed (Table 4). Saudi Arabia’s model shares structural similarities with the UK’s centralized approach, with top-down implementation driven by a national agenda. Both nations rely on a unified digital backbone, the NHS Spine in the UK and Sehhaty/Wasfaty in Saudi Arabia. However, a critical divergence lies in regulatory maturity. While the UK has established rigorous, standardized governance frameworks for remote services like the NMS, Saudi Arabia currently faces regulatory ambiguity, with inconsistent enforcement of online pharmacy standards [12]. In contrast, the USA operates on a decentralized model, where innovation is often driven by the private sector and regulated at the state level, creating a patchwork of high-maturity pockets that lacks the uniformity of the UK or Saudi systems.

**Table 4:** Comparative analysis of telepharmacy models MOH: Ministry of Health; NHS: National Health Service; EPS: Electronic Prescription Service; MTM: medication therapy management; NMS: New Medicine Service; DMS: Discharge Medicines Service; BP: blood pressure

Feature	Saudi Arabia (Emerging)	United States (Mature - Decentralized)	United Kingdom (Mature - Centralized)
Governance model	Centralized (MOH-led): Developing regulation; variable awareness and enforcement of standards [12].	Decentralized (state-level): Regulated by State Boards; mature but variable across states [16].	Centralized (NHS-led): Unified governance with standardized national scopes and frameworks [3].
Key infrastructure	National platforms: Sehhaty & Wasfaty provide a unified digital base [4, 7].	Private sector: Highly interoperable electronic health records and clinical decision support systems [2].	National backbone: NHS Spine and EPS enable nationwide connectivity [3].
Reimbursement	Government-funded: Reimbursement pathways are mixed or expanding; private insurance models are developing [5].	Fee-for-service: Established reimbursement for services like MTM [17].	Commissioned services: NHS-funded services commissioned nationally (e.g., NMS, DMS) [25, 26].
Workforce readiness	Willing but unprepared: Pharmacists are willing but face training and competency gaps [11].	Broad experience: Telehealth is embedded in practice with broad pharmacist experience [3].	Structured Roles: Pharmacists have structured roles within established NHS pathways [26].
Evidence base	Early outcomes: Evidence limited to diabetes and anticoagulation [8, 9].	Extensive: Strong evidence for adherence, BP control, and glycemia [22].	Strong: High evidence for implementation success and service integration [29].

Financial sustainability and reimbursement: The most significant disparity between the regions is the financial model supporting telepharmacy. In both the USA and UK, telepharmacy is a reimbursable service, ensuring its financial sustainability. The USA utilizes specific billing codes for MTM, incentivizing pharmacists to provide cognitive services remotely [17]. Similarly, the UK commissions specific remote services like the NMS [25]. In Saudi Arabia, the model remains largely government-funded and product-centric; there is currently no clear mechanism for private community pharmacies to bill insurers for "virtual consultations" independent of dispensing a product. This economic gap limits the expansion of telepharmacy from a government initiative to a viable private-sector business model.

Workforce readiness and operational maturity: Operational maturity also varies significantly. In the USA and UK, telepharmacy workflows are well-integrated into routine practice, supported by specialized training and defined roles for pharmacy technicians as demonstrated in U.S. remote dispensing models and UK rural telepharmacy services [16]. In Saudi Arabia, while the JHAH model demonstrates that high-maturity operations are possible [10], the broader landscape is characterized by a readiness-practice gap. Although Saudi pharmacists report high willingness to adopt digital and remote-care technologies, they lack the specific training and standardized operating procedures prevalent in the comparator nations [11].

Discussion

The Infrastructure-Operation Gap

This review highlights a distinct paradox in Saudi Arabia’s telepharmacy landscape. While the Kingdom has achieved rapid digital infrastructure deployment comparable to the UK’s NHS, evidenced by national platforms like Sehhaty and Wasfaty, its operational maturity lags behind. Unlike the USA, where telepharmacy is a commercially viable, reimbursed service integrated into routine care, Saudi Arabia’s model remains largely functional and logistical, primarily focused on e-prescribing rather than cognitive pharmaceutical care. The North Dakota and NHS NMS models demonstrate that technology is merely an enabler; the true driver of telepharmacy success is a supportive regulatory and financial ecosystem, which is currently underdeveloped in the Kingdom [16,25]. This operational maturity in the UK is further reflected in medication-focused remote care models for chronic conditions, such as COPD, as well as rural telepharmacy proof-of-concept services [29,30].

Regulatory Ambiguity and Economic Barriers

A primary barrier identified is the lack of specific, enforceable regulations for remote pharmaceutical care. While general telemedicine guidelines exist, specific protocols for virtual pharmacist consultations are ambiguous compared to the rigorous standards of the General Pharmaceutical Council in the UK. Furthermore, the economic model presents a significant hurdle. In the USA, the existence of CPT billing codes for MTM incentivizes private pharmacies to invest in telepharmacy infrastructure [17]. In Saudi Arabia, the absence of such reimbursement mechanisms limits telepharmacy adoption to government-funded initiatives or large institutional outliers like JHAH, stifling private sector innovation and scalability [12].

Workforce Readiness: The Willingness-Competency Mismatch

Our comparison reveals a critical workforce gap. Saudi pharmacists express a high willingness to adopt digital tools, yet they report a lack of standardized training in virtual communication and digital ethics [11]. This stands in contrast to the USA, where telepharmacy and telehealth competencies are increasingly incorporated into PharmD curricula through didactic instruction, simulation-based training, and experiential education, reflecting national efforts to prepare graduates for remote patient-care roles [24]. Without targeted upskilling, the transition from traditional dispensing to remote patient management will remain slow, regardless of technological availability.

Operational Challenges in National Platforms

While Wasfaty has successfully digitized prescribing, its operational limitations, specifically stock visibility and lack of integrated safety alerts, disrupt the continuity of care [7, 14]. In the UK’s EPS system, a patient can be virtually directed to a specific pharmacy with guaranteed stock, ensuring a seamless loop [3]. In Saudi Arabia, the disconnect between the digital prescription and the physical inventory forces patients to physically navigate multiple pharmacies, negating the convenience promised by telepharmacy.

Limitations

This review is limited by the scarcity of large-scale longitudinal outcome studies in Saudi Arabia. Most local literature relies on cross-sectional surveys of perception rather than rigorous clinical trials, making direct comparison with the robust clinical evidence base of the USA and UK challenging.

## Conclusions

Saudi Arabia stands at a pivotal moment in advancing telepharmacy for chronic disease management. Under Vision 2030, the Kingdom has successfully laid a robust digital infrastructure capable of supporting remote care for conditions such as diabetes and anticoagulation disorders, as demonstrated in early clinical pilots. However, this review confirms that digital infrastructure alone is insufficient to achieve the level of maturity seen in the USA and the UK. To move from basic digital prescribing toward comprehensive telepharmacy that delivers evidence-based long-term care comparable to international standards, Saudi Arabia must complement its technological investments with strong regulatory, financial, and educational frameworks. Evidence from established telepharmacy models in the USA and UK indicates that sustainable remote pharmaceutical care depends on clear governance structures, robust reimbursement mechanisms, and standardized clinical workflows tailored to therapeutic monitoring and patient counseling.

To optimize health outcomes and ensure telepharmacy becomes a cornerstone of the Kingdom’s chronic care strategy, coordinated action is required across multiple domains. Regulators should develop specific licensure standards and clearly defined scopes of practice for telepharmacy providers, particularly for remote consultations in chronic disease management. Payers, including the Council of Cooperative Health Insurance, need to introduce reimbursement mechanisms for pharmacist-led virtual consultations, similar to medication therapy management models, to incentivize scalable, sustainable services. Pharmacy education should embed digital health and telepharmacy as core components of undergraduate and postgraduate training, ensuring pharmacists are prepared for evidence-based remote care. Finally, national platforms such as Wasfaty should evolve beyond prescription transmission to incorporate real-time inventory visibility and clinical decision support, enabling drug-drug interaction checking, dosing optimization, and structured patient education. Together, these integrated reforms can position Saudi telepharmacy as a sustainable and scalable model that improves chronic disease control, enhances medication adherence, and reduces complications and hospitalizations across the Kingdom.
